# Percutaneous Cholecystostomy in Severe Acute Cholecystitis: An Observational Study From a Single Institute

**DOI:** 10.7759/cureus.34539

**Published:** 2023-02-02

**Authors:** Kirolos Abdelsaid, Mohamed Hassan, Balaji Jayasankar, Milad Jeilani, Haythem Ali, Yasser Abdul Aal

**Affiliations:** 1 General Surgery, Maidstone and Tunbridge Wells NHS Trust, Tunbridge Wells, GBR; 2 Surgery, Maidstone and Tunbridge Wells NHS Trust, Tunbridge Wells, GBR; 3 Gastrointestinal Surgery, Maidstone and Tunbridge Wells NHS Trust, Tunbridge Wells, GBR

**Keywords:** tokyo guidelines, post cholecystectomy complications, acute calculus cholecystitis, laparoscopic cholecystectomy, percutaneous cholecystostomy tube

## Abstract

Background

Although percutaneous cholecystostomy (PC) is generally accepted as a bridge to definitive therapy for acute cholecystitis (AC), which remains cholecystectomy, some patients did not undergo cholecystectomy, mainly due to contraindications to surgery. Here, we aimed to investigate the predictors of recurrence and the outcome after PC.

Methods

This is a retrospective study from a single general hospital at Tunbridge Wells, United Kingdom. One hundred twenty-six patients who presented with AC grade 3 and were initially managed with PC were included. In addition, the proportion of patients who did not undergo subsequent laparoscopic cholecystectomy (LC) and their characteristics were analyzed.

Results

The mean age of the study cohort was 72 (36-98) years, and the median length of drain insertion was 39.5 days. The majority (52%) presented with severe AC grade 3 with failed medical treatment to control the disease, while 7% had an emphysematous gallbladder. Eighty percent of patients did not develop any further attacks of AC after PC removal. The most common comorbidity was hypertension (35%). The mean age-adjusted Charlson comorbidity score was 3.72. Thirty-six percent (45/126) of the study cohort underwent LC, while the remaining patients did not receive any surgical intervention. Nine percent were deemed unfit for surgery. Forty-one patients (33%) were managed conservatively as they did not have a further attack of cholecystitis after PC removal or had a mild attack managed with antibiotics. In addition, 22% experienced procedural complications, including a blocked stent, pain, and cellulitis around the tube. The 30-day mortality rate of patients who did not undergo LC was 5%. Predictors of interval cholecystectomy were younger age, calculus cholecystitis, low Charslson index score, and uncomplicated and shorter length of hospital stay with PC.

Conclusion

Most severe AC patients treated initially with PC did not undergo subsequent LC. Therefore, PC in high-surgical-risk patients with AC could be a definitive treatment.

## Introduction

Gallbladder disease is a common GI disorder. Approximately 15% of the United Kingdom (UK) adult population is thought to have cholelithiasis. Twenty percent of people with cholelithiasis are symptomatic. About 50,000 cholecystectomies are performed each year in the UK, about one-third of which are for cholecystitis [1]. According to Tokyo guidelines, acute cholecystitis (AC) is classified into three grades [2]. Grade 3 AC is associated with organ system dysfunction, so it is crucial not to expose the patient to further surgical trauma in the form of cholecystectomy unless they are fit for surgical intervention. Therefore, the guidelines recommend supportive care and urgent radiological gall bladder drainage [3]. Early laparoscopic cholecystectomy (LC) within a week of the acute symptoms is the standard treatment method for patients who are fit for surgery [4]; however, cholecystectomy could be risky in patients with other critical comorbidities. Percutaneous cholecystectomy (PC) provides an effective, minimally invasive alternative and safe procedure to manage patients with acute cholecystitis who are critically ill [5] and deemed unfit for surgery or have no appreciable clinical improvement after initial antibiotic therapy. In acute calculous cholecystitis, although PC is generally accepted as a bridge to definitive therapy, which remains cholecystectomy, some patients do not undergo cholecystectomy after gallbladder drainage, most often due to ongoing contraindications to surgery [6]. In addition, sometimes, patients do not present with any further attacks of AC requiring surgical intervention or mild attacks of AC, which can be managed conservatively. Therefore, elective LC should be offered to patients once cholecystitis resolves and they become fit for surgery. There is no clear consensus in managing the patient with severe AC who has been treated initially with PC either in the timing of cholecystectomy or the selection criteria for cholecystectomy after gall bladder drainage. In this study, we aimed to conduct an observational study from a single institution to determine the PC effectiveness as definitive management for patients with AC. Therefore, we looked at the patients’ characteristics and radiological findings that led to PC and the associated morbidity and mortality rates.

## Materials and methods

Data for all the patients who underwent PC for acute severe cholecystitis (Tokyo grade 3) or complicated AC who did not improve on initial antibiotics therapy from January 2016 to January 2021 at Maidstone and Tunbridge Wells NHS Trust, a single general district hospital, UK, were retrieved retrospectively. The patients' demographics included age at the time of presentation, gender, and age-adjusted Charlson Index Score. In addition, laboratory investigations, including white cell count (WCC), C-reactive protein (CRP), total bilirubin (TB), and alkaline phosphatase (ALP), were collected as a predictor of either recurrence or interval cholecystectomy. The indications and the radiological findings for PC were noted. These include severe AC with failed medical treatment, perforated gall bladder, empyema of the gall bladder, or associated abscess. Primary outcomes included hospital length of stay, 30-day mortality, intensive care admission, 30-day re-admission, another attack of cholecystitis, procedural complications, biliary stent insertion either radiologically or endoscopically, duration of PC insertion, the timing to interval cholecystectomy, and the PC outcomes. Secondary outcomes included cholecystectomy and recurrence of cholecystitis.

Statistical analysis

SPSS version 26, for Windows, was used to carry out all statistical analyses. Cohort characteristics were performed, including frequency and distribution of the various parameters. The Chi-square test was used to assess the association between categorical parameters and the outcomes, while Mann-Whitney was used in continuous data. Multivariate logistic regression analysis was carried out. P value <0.05 was considered statistically significant. The local ethical committee at Maidstone and Tunbridge Wells NHS Trust, UK (approval #1418) has approved this study. The data used was fully anonymized. We acknowledge that this study complies with the Helsinki declaration for research.

## Results

Over the study period, 126 patients underwent PC. Sixty-six patients (52%) were males. The mean age at the time of diagnosis was 72 years. The most common comorbidities were hypertension and cardiac diseases, 44 (35%) and 35 (28%) patients, respectively. The mean age-adjusted Charlson comorbidity score was 3.72. Sixty-six (52%) patients had severe AC (Tokyo grade 3) with failed medical treatment, while perforated gallbladder was seen in 21 patients (17%). Eight-six (73%) patients had calculous cholecystitis. The median WCC was 15.5*10^9/L. Thirty-two (25%) patients had previous abdominal surgery. Table 1 summarises the patients' demographics and characteristics. 

**Table 1:** Cohort characteristics.

Parameter	Number/Percentage
Gender	
Male	66 (52)
Female	60 (48)
Age at diagnosis (Year)	
≥ 60	100 (79)
< 60	26 (21)
Indication of Percutaneous cholecystostomy	
Acute severe cholecystitis (Tokyo grade 3) with failed Medical treatment	66 (52)
Perforated Gallbladder	21 (17)
Gallbladder empyema	19 (15)
Acute cholecystitis with liver abscess	11 (9)
Emphysematous Gallbladder	9 (7)
Type of cholecystitis	
Calculous	86 (68)
Acalculous	32 (32)
Comorbidities	
Heart disease	35 (28)
Pulmonary disease	20 (16)
Neurological disorders	21 (17)
Hypertension	44 (35)
Diabetes	23 (18)
Malignancy	19 (15)
Blood investigations	
White cell count	15.5 (1-45) *10^9/L
C -eactive protein	248.5 (21-543) mg/L
Total Bilirubin	16 (5-173) umol/L
Alkaline phosphatase	149 (16-1029) U/L
Previous Abdominal Surgery	
Yes	32 (25)
No	94 (75)

The median duration of having PC was 39.5 days (range 1-220 days), while the median hospital length of stay (defined as the date of admission till the date of discharge) was six days (range 1-63 days). Seven percent of the patients were admitted to the ICU. Three patients (2%) were readmitted within 30 days of discharge, and the 30-day mortality rate was 5%. Twenty-eight (22%) patients had periprocedural complications, including dislodgment of the tube (12%). Only three patients had their dislodged tubes replaced, and the rest of the patients managed conservatively without the need for further insertion. Seven patients had blocked drains managed by flushing the drain using normal saline in an emergency outpatient clinic. Three patients had cellulitis, and the subcutaneous collection was managed using antibiotics. Two patients had a subphrenic collection that required radiological drainage, and the patient who had acute kidney injury from high-output PC was treated with IV fluid. Thirteen (10%) patients had a biliary stent inserted either radiological or endoscopically. A total of 20% of the patients had a recurrent attack of AC requiring hospital readmission. Among the patients with recurrence, only two had functional PC at the time of diagnosis. The remaining patients had PC removed. A total of 11 (9%) patients had recurrent AC more than 90 days after discharge. Forty-five patients (36%) underwent interval cholecystectomy (87% LC and 13% either converted to open or was open cholecystectomy from the start). Forty-one (33%) patients who had regular follow up in the outpatient clinic were managed conservatively. A total of 6/41 (15%) patients managed conservatively had recurrent cholecystitis and were managed with broad-spectrum antibiotics. The rest of the patients were reviewed in the outpatient clinic. The median hospital length of stay post cholecystectomy was a 1-day range (0-12 days). In addition, 20% of the patients had postoperative complications from wound infection to postoperative collection managed with a radiological drain. Table 2 summarises the various outcomes of patients with PC placement.

**Table 2:** Outcomes of patients with percutaneous cholecystostomy tube placement. *Includes (one high drain output acute kidney injury, two subphrenic collections) ** These parameters are presented with the median and the range ^***^ Endoscopic retrograde cholangiopancreatography **** Length of stay ^***** ^Intensive therapy unit

Outcome	Number/Percentage
30-day mortality	7 (5)
30-day readmission	3 (2)
30-60-day readmission	10 (8)
60-90-day readmission	1 (0.7)
> 90-day readmission	11 (9)
Recurrent Cholecystitis	25 (20)
Percutaneous cholecystectomy status at the time of recurrence	
Removed	23
Functional	2
Biliary stent, either radiological or ERCP^***^	13 (10)
ERCP after removal of percutaneous cholecystostomy	6
Radiological cystic duct stent	4
ERCP, percutaneous cholecystostomy still insitu	3
Procedural complications	28 (22)
Dislodgment	15 (12)
No reinsertion	12
Reinserted	3
Blocked drain	7
Subcutaneous collection or cellulitis	3
Others*	3
ITU***** admission	9 (7)
The outcome of percutaneous cholecystostomy	
Surgery	45 (36)
Conservative management	41 (33)
Died within 90 days	12(10)
Not fit for surgery	11 (9)
Awaiting preoperative assessment	9 (7)
Refuse surgery	7 (6)
Moved to other countries	1
30-day mortality after cholecystectomy *	0 (0)
Cholecystectomy	
Laparoscopic	39 (87)
Open	6 (13)
Complications from cholecystectomy	9 (20)
Hospital LOS^****^ with percutaneous cholecystostomy **	6 (1-63) days
Duration of percutaneous cholecystostomy insertion **	39.5 (1-220) days
Duration from percutaneous cholecystostomy insertion to cholecystectomy **	144 (31-757) days
Duration from percutaneous cholecystostomy removal to cholecystectomy**	86 (0-714) days
LOS post cholecystectomy **	1 (0-12) days

Predictors of recurrence 

Detailed univariate comparisons of patients with recurrence versus no recurrence are listed in Table 3. Analysis revealed that a high WCC (p=0.03) at the time of tube insertion was associated with a higher probability of recurrence of AC. Patients with periprocedural complications did not have significantly lower odds of recurrence (p=0.117). Neither length of drain insertion nor the PC indication was correlated with recurrence (p=0.328 & 0.472, respectively).

Predictors of interval cholecystectomy 

Detailed univariate comparisons of patients with interval cholecystectomy versus no interval cholecystectomy are listed in Table 3. Patients with acalculous cholecystitis (p<0.001) were less likely to have a cholecystectomy. We noticed a statistical significance toward cholecystectomy for patients with shorter hospital lengths of stay with PC (p=0.007). In addition, there was statistical significance for the younger patients (p<0.001) and low Charlson index (p<0.001). Patients with periprocedural complications (p=0.010) were more likely to undergo interval cholecystectomy. Multivariate logistic regression analysis showed calculus cholecystitis, uncomplicated tube, and low Charlson index were independent predictors for interval cholecystectomy regardless of the other factors (p=0.009, p=0.016, and p=0.003, respectively).

**Table 3:** Predictors of interval cholecystectomy and recurrence. ^* ^These parameters are presented with the median and the range ^** ^Intensive therapy unit ^*** ^Length of stay

Parameter	Surgery performed			Recurrent cholecystitis		
	Yes (N, %)	No (N, %)	P-value	Yes (N, %)	No (N, %)	P-value
Gender						
Male	20 (30)	46 (70)	0.160	14 (21)	52 (79)	0.950
Female	25 (42)	34 (58)		13 (22)	47 (78)	
ITU^**^ admission						
Yes	3 (33)	6 (67)	0.863	2 (22)	7 (78)	0.952
No	42 (36)	74 (64)		25 (21)	92 (79)	
Previous abdominal surgery						
Yes	7 (22)	25 (78)	0.054	9 (28)	23 (72)	0.285
No	38 (41)	55 (59)		18 (19)	76 (81)	
Tube complications						
Yes	4 (15)	23 (85)	0.010	9 (32)	19 (68)	0.117
No	41 (42)	57 (58)		18 (18)	80 (82)	
Percutaneous cholecystostomy indication						
Acute cholecystitis with liver abscess	2 (18)	9 (82)	0.373	3 (27)	8 (73)	0.472
Perorated Gallbladder	9 (43)	12 (57)		4 (19)	17 (81)	
Emphysematous Gallbladder	2 (22)	7 (78)		4 (44)	5 (56)	
Acute cholecystitis with failed medical treatment	23 (35)	43 (65)		12 (18)	54 (82)	
Gallbladder Empyema	9 (50)	9 (50)		4 (21)	15 (79)	
Type of cholecystitis						
Acalculous	4 (13)	28 (87)	<0.001	7 (22)	25 (78)	0.911
Calculous	39 (46)	46 (54)		18 (21)	68 (79)	
Biliary stent						
Yes	6 (43)	8 (57)	0.587	5 (36)	9 (64)	0.173
No	39 (36)	71 (64)		22 (20)	89 (80)	
Age*	72 (42.00-74.81)		<0.001	72 (60.63-64.28)		0.645
Charlson index*	4 (36.99-77.63)		<0.001	4 (65.15-57.46)		0.327
LOS^***^ with perutaneous cholecystostomy*	6 (51.38-69.54)		0.007	6 (62.86-65.85)		0.705
Length of drain insertion*	39.5 (65.32-55.91)		0.147	39.5 (57.86-65.29)		0.328
White Cell Count	15.5 (64.14-62.36) *10^9/L		0.791	15.5 (59.82-76.98) *10^9/L		0.03
C-Reactive Protein	248.5 (67.83-60.28) mg/L		0.263	248.5 (63.99-61.70) mg/L		0.773
Alkaline Phosphatase	149 (58.32-65.63) U/L		0.279	149 (62.75-66.26) U/L		0.658
Total Bilirubin	16 (60.39-64.47) umol/L		0.545	16 (61.87-69.48) umol/L		0.336

## Discussion

It has been proven that early LC is the preferred option for managing AC in patients fit for surgery [7,8]. The 2013 Tokyo guidelines [3] state that immediate biliary drainage via PC should be performed among high-risk patients with grade II (moderate) and III (severe) AC. PC reduces inflammation of the gallbladder and the risk of septicemia [9]. It is also proposed as a definitive treatment in high-risk surgical patients [10]. To the best of our knowledge, this study is unique in addressing several variables related to using PC in severe AC. The outcome post-PC insertion was one of these essential variables yet to be discussed in the literature. All the patients have been followed up in the outpatient clinic and assessed by upper GI surgeons and anesthetists. Forty-one patients (33%) have been managed conservatively without surgical intervention as they have not had a further attack of cholecystitis after PC removal or biliary colic or have a mild attack and are managed with antibiotics. The unique findings about our cohort are the identification and follow-up of the patients managed conservatively and/or unfit for surgical intervention, as their comorbidities were still significant at a median follow-up duration of 3.1 (1.2-6.5) years. The decision of conservative management was a shared decision between the patients and surgeon, which was to continue to wait and watch, and if there was any improvement in the patient's comorbidity, surgeons would proceed with the surgical management. Eleven patients (9%) were deemed unfit for surgical intervention during the follow-up as their comorbidities were still significant. This cohort's 30-day mortality was 5%, marginally less than other recently large single-center studies [11,12]. The main reason for the low mortality rate is the multidisciplinary management protocols for such patients; this team includes surgeons, interventional radiologists, intensivists, microbiologists, and trained nursing staff who provide regular follow-ups.

PC is considered a safe and effective alternative treatment option for severe AC in patients with significant comorbidity; however, it might be associated with several complications, likewise any surgical procedure [13]. The rate of complications, including dislodgment, blocked drain, and cellulitis, was 22%, less than the complications reported in different literature [13]. In addition, all patients with PC received pamphlets explaining how to flush the tube, the complication that might happen, and all the required information regarding percutaneous cholecystostomy. The leading cause of readmission was a recurrent attack of cholecystitis (19.8%) at a median of 76 days. Twenty-three patients had a recurrent attack after PC removal, and only two patients had an AC while the PC was functioning. The readmission rate between 60 and 90 days in our study was consistent with the recurrence rate of another study [14]. Interval cholecystectomy post PC insertion for patients with AC is frequently deferred in high-risk surgical patients. Once the patient is in a fit state for general anaesthesia, the surgery should be offered to this group of patients. The rate of interval cholecystectomy from the removal of PC in our cohort was slightly higher than described by Horn T et al. [14-15]. This can be explained by the fact that all the patients in our study who had a PC inserted during the index of admission had not had cholecystectomy during the same admission. PC in high-surgical-risk patients with AC could be a definitive treatment [16,17]. The limitations of this study include its retrospective design and some factors or variables that may not be investigated that were the reasons for the difference in outcomes. The relatively small sample size and shorter follow-up period to perform precision statistical analysis to answer the critical question is what the parameter of using PC as a definitive treatment for AC grade 3 is. The multidimensional criteria for interval cholecystectomy or conservative management (clinical, social, and surgeon decision) weakened the study as there were no precise guidelines to follow.

## Conclusions

In conclusion, most severe AC patients treated initially with PC did not undergo subsequent LC. PC is considered an effective, less minimally invasive procedure in treating AC grade 3 and could be a definitive treatment in high-risk patients. Future studies are needed to identify the exact parameters for using PC as a definitive treatment for AC grade 3.
